# Outcomes of alternative therapy in HLA-B* 13:01 positive leprosy patients without dapsone versus standard MDT in negative patients: A comparative effectiveness study

**DOI:** 10.1371/journal.pntd.0014114

**Published:** 2026-03-17

**Authors:** Yang Li, Zhenzhen Wang, Tongsheng Chu, Hong Wang, Lijiao Yin, Shujuan Yuan, Yonghong Wang, Gang Li, Hong Liu, Furen Zhang

**Affiliations:** 1 School of Public Health, Cheeloo College of Medicine, Shandong University, Jinan, Shandong, China; 2 Dermatology Hospital of Shandong First Medical University, Jinan, Shandong, China; 3 Shandong Provincial Institute of Dermatology and Venereology, Shandong Academy of Medical Sciences, Jinan, Shandong, China; 4 Guangxi Provincial Institute of Dermatology, Nanning, Guangxi, China; 5 Wenshan Prefecture Dermatology Prevention and Treatment Institute (Wenshan Prefecture Dermatology Specialist Hospital), Wenshan, Yunnan, China; 6 Hunan Provincial Center for Disease Control and Prevention (Hunan Academy of Preventive Medicine), Changsha, Hunan, China; 7 Xingyi Institute of Dermatology and Venereology, Xingyi, Guizhou, China; 8 Congjiang County Center for Disease Control and Prevention, Congjiang, Guizhou, China; 9 School of Public Health, Shandong First Medical University, Jinan, Shandong, China; Colorado State University, UNITED STATES OF AMERICA

## Abstract

**Background:**

In HLA-B*13:01-positive multibacillary (MB) leprosy patients, dapsone-containing multidrug therapy (MDT) carries a high risk of dapsone hypersensitivity syndrome (DHS). Alternative regimens (dapsone-free) are adopted, but their long-term efficacy compared with standard MDT in HLA-B*13:01-negative patients remains inadequately characterized.

**Methodology:**

This retrospective cohort study analyzed MB patients (2015–2023) from the National Leprosy Prevention and Control Management Information System (LEPMIS) with ≥1-year follow-up. Primary outcomes (cure/relapse rates, bacterial index (BI), leprosy reactions, and disability progression) and secondary outcomes (adverse events and treatment duration) were compared between HLA-B*13:01-positive patients receiving alternative therapy (rifampicin + clofazimine ± clarithromycin/ofloxacin/minocycline) and negative patients receiving standard MDT (rifampicin + clofazimine + dapsone).

**Findings:**

Among the 271 enrolled MB patients (120 HLA-B*13:01-positive, 151 negative), alternative therapy showed comparable efficacy to standard MDT in cure rates (67.6% vs. 65.8% at Year 5), the rate of BI decline (89.92% vs. 95.11% at Year 5), smear negativity rates (71.43% vs. 75.00% at Year 5) and relapse rates (0.46 vs. 0.20 per 100 person-years). Kaplan-Meier survival functions revealed no significant differences in leprosy reactions or disability progression. Additionally, alternative therapy demonstrated comparable safety to MDT (1.67% vs. 2.65%, *P* = 0.70).

**Conclusions:**

In our study, dapsone-free alternative regimens demonstrated comparable clinical efficacy and safety to standard MDT in MB patients, providing a viable option for HLA-B*13:01 carriers. These findings, limited by the observational design and regimen heterogeneity, warrant further investigation in prospective trials.

## Author summary

Leprosy remains a major global health challenge. Although WHO-recommended multidrug therapy (MDT) is the standard regimen, the component dapsone (DDS) may cause fatal dapsone hypersensitivity syndrome (DHS). Pretreatment HLA-B*13:01 screening identifies carriers, enabling DDS-free regimens to reduce DHS risk; however, whether their efficacy matches standard MDT remains unclear. This study compared the long-term effectiveness and safety of DDS-free regimens versus standard MDT in multibacillary (MB) leprosy patients, providing critical evidence for clinical decision-making. In a retrospective cohort of 271 MB patients (120 HLA-B*13:01-positive, 151 negative) from National Leprosy Prevention and Control System (2015–2023), we assessed cure/relapse rates, bacterial index (BI) change, leprosy reactions, disability progression, and adverse events. Alternative regimens (rifampicin + clofazimine ± clarithromycin/ofloxacin/minocycline) demonstrated comparable efficacy and safety to standard MDT, despite numerically lower BI decline rates and a non-significantly longer mean treatment duration (1.68 vs. 1.60 years, *P* > 0.05), providing a viable option for HLA-B*13:01 carriers. This study offers critical real-world evidence to optimize leprosy management in genetically susceptible populations, aligning with global efforts to eliminate DHS risks while maintaining therapeutic outcomes.

## Introduction

Leprosy, or Hansen’s disease, is a chronic infectious disease caused by *Mycobacterium leprae*, primarily affecting the skin and peripheral nervous system. It presents with diverse clinical manifestations, prolonged incubation periods, and significant transmissibility. Without timely intervention, it can lead to irreversible disabilities, imposing substantial physical, psychological, and socioeconomic burdens on individuals and communities [1]. In the 21st century, global leprosy incidence has not declined significantly, with over 200,000 new cases reported annually worldwide and it remains a critical public health concern for governments across nations [2].

Dapsone (4,4’-diaminodiphenylsulfone, DDS), one of the core components of multidrug therapy (MDT), plays a critical role in leprosy treatment through its bacteriostatic effects on *M. leprae*. It inhibits bacterial DNA synthesis by interfering with folate metabolism and also exhibits anti-inflammatory properties [3–5]. However, its use is associated with a severe adverse drug reaction known as dapsone hypersensitivity syndrome (DHS), which affects 0.5–3.6% of treated patients [6,7]. DHS typically presents with fever, rash, and systemic organ involvement, and carries a mortality rate of 9.9–12.5% [8]. In 2013, genome-wide association studies identified HLA-B*13:01 as a major genetic risk factor for DHS [6]. In response, China initiated nationwide pretreatment HLA-B*13:01 screening in 2015 across 21 provinces, allowing avoidance of dapsone in carriers and virtually eliminating DHS in this population [9].

The removal of dapsone from standard MDT raises concerns about whether alternative regimens maintain equivalent efficacy. Several studies have explored this issue. A retrospective study in Nepal (1990–2007) evaluated modified multidrug therapy following dapsone adverse effects [10]. Similarly, a study in Guangxi, China, assessed dapsone-free alternative regimens in HLA-B*13:01-positive multibacillary (MB) patients versus conventional MDT regarding bacterial index (BI) [11]. Additionally, a randomized controlled trial (RCT) in India compared paucibacillary (PB) multidrug therapy with the rifampicin-ofloxacin-minocycline (ROM) regimen for cure and relapse [12]. A recent small parallel clinical trial also compared the ROM regimen with WHO-MDT for MB patients [13].

However, previous studies still exhibit limitations, including restricted sample sizes and narrow efficacy metrics [10–14]. To systematically evaluate whether DDS-free alternative regimens are comparable in efficacy to standard MDT, we established a real-world retrospective cohort to compare alternative therapy for HLA-B*13:01-positive leprosy patients with WHO-MDT. Our multidimensional assessments encompassed cure/relapse rates, BI change, leprosy reactions, disability progression, and adverse drug reactions. This will provide an evidence base for optimizing MDT strategies, particularly for HLA-B*13:01 carriers. Furthermore, since the enrolled patients were predominantly MB leprosy, this study mainly analyzed this population.

## Methods

### Study design

This study adheres to the STROBE guidelines for reporting observational cohort studies (S1 Checklist) [15]. Based on prior HLA-B*13:01 testing research, we classified MB leprosy patients as positive or negative using pretreatment genetics. By March 2025, 4,380 newly diagnosed patients were enrolled (758 positive; 3,622 negative). We initially stratified positives by province, forming a positive cohort from provinces with ≥10 positive cases. Age- and sex-matched negative controls were selected via quota sampling from corresponding provinces, with the goal of identifying one to two controls per positive patient.

Using the National Leprosy Prevention and Control Management Information System (LEPMIS), we conducted a retrospective follow-up survey. The study aimed to include all eligible MB leprosy cases meeting inclusion criteria during 2015–2023 to ensure adequate representation of both HLA-B*13:01-positive and negative cohorts. Positive patients received alternative DDS-free therapy (Alternative Group); negative patients received standard MDT (rifampicin + clofazimine + dapsone; MDT Group). The specific alternative treatment regimens adopted in this study can be divided into two main categories: 1) Rifampicin (RFP) 600mg/month supervised + clofazimine (B663) 300mg/month supervised and 50mg/day self-administered); 2) Replace DDS with clarithromycin (500mg daily)/ofloxacin (400mg daily)/minocycline (100mg daily) (COM), and the rest with MDT.

Our primary outcomes included bacteriological indicators (the rate of BI decline, smear negativity rate), cure/relapse rates, leprosy reaction frequency and disability progression (DP), with safety evaluation and treatment duration as secondary outcomes. Data were collected using a standardized questionnaire. Annual follow-up commenced at treatment initiation, with ≥1 year follow-up.

### Outcome definitions and measurements

Cure and relapse were defined according to the Chinese leprosy diagnostic standard (WS 291–2018) [16]. Specifically, cure was defined as the disappearance of all active clinical symptoms with no evidence of leprosy reactions or neuritis, confirmed by negative bacteriological tests on three consecutive occasions at intervals of >3 months. Relapse was defined as the reappearance of clinical signs of disease activity in a patient previously considered cured, supported by bacteriological or histopathological evidence. The comprehensive definitions for these outcomes (e.g., cure, relapse, DP) are provided in S1 Text.

The BI was assessed following the same standard [16]. For each patient, slit‑skin smears were taken from 4-6 sites, including routine areas (e.g., supraorbital, earlobes, chin) and 2–3 of the most active lesions. Smears were Ziehl-Neelsen stained, examined under oil immersion, and graded using the Ridley logarithmic scale; the BI was calculated as the mean score across all sites. All readings were performed by certified technicians at designated provincial reference laboratories, which participate in annual external quality assurance programs to ensure consistency and accuracy. The rate of BI decline was calculated at the individual level using the formula: (Follow-up BI - Baseline BI)/ Baseline BI * 100%. For group comparisons, the mean of these individual rates was used as the summary measure.

Data on adverse drug reactions (ADRs) were retrospectively extracted from the LEPMIS. The ADR records and their severity gradings were initially assessed and documented by clinicians based on clinical manifestations and laboratory findings according to routine diagnostic practice. Additionally, two experienced investigators independently applied the WHO-UMC causality criteria to perform final confirmation and consistency checks for all ADR cases [17].

### Sample size

A sample‑size calculation was performed to ensure adequate power. Based on an approximate 1:1.5 group ratio and assuming a 3‑year cure rate of 50% for MDT, with an absolute difference of 20% in favor of MDT, a two‑sided alpha of 0.05, 80% power, and 10% loss to follow‑up, the minimum required sample size was 86 in the alternative group and 129 in the MDT group. Our final analyzable sample sizes exceeded these minima, indicating sufficient power for the comparison.

### Inclusion/exclusion criteria of patients

We selected the MB leprosy patients from all provinces who were sent to Shandong Dermatological Hospital for HLA-B*13:01 gene testing from 2015 to 2023. Inclusion criteria: a) Untreated newly diagnosed MB leprosy patients or relapsed cases (with last treatment >5 years before study enrolment) [18]; b) All aged 6–70 years [12]. c) Complete bacteriological data: standardized BI testing of interstitial fluid at baseline and annually thereafter, with follow-up measurements accepted within a ± 2-month window of each yearly anniversary of treatment initiation [13,19]. Exclusion criteria [12,20]: a) Suffering from tuberculosis, severe lung disease, bronchial asthma, significant acquired immunodeficiency syndrome disorder, hepatic or renal dysfunction, or current treatment with steroids; b) Pregnant and breastfeeding women; c) Inadequate adherence to standardized treatment or follow-up care; d) Incomplete bacterial index records; e) Follow-up duration <1 year.

### Statistical analyses

Data were analyzed using IBM SPSS Statistics (27.0.1; IBM Corp.) and R (4.3.3; R Foundation for Statistical Computing). For descriptive statistics, continuous variables were reported as mean (±SD) or median; categorical variables as counts (%). Group comparisons employed Pearson’s *χ²* (frequencies) and independent t-tests/Mann-Whitney U (BI decline rate, treatment duration). Multiple comparisons were corrected using the Bonferroni method. Relapse rates were compared using Poisson regression, modeling relapse counts with treatment group (Alternative therapy vs. MDT) as the predictor and log (person-years) as an offset. Results are presented as rate ratios (RR) with 95% confidence intervals (CIs) and two-sided p-values. Fitting curves for BI decline rates were generated using polynomial regression. A Cox proportional hazards model was fitted to estimate the treatment effect on time to cure, adjusting for potential confounders (age, sex, disease duration, and initial BI). Kaplan-Meier curves with log-rank tests evaluated time-to-event outcomes (first reaction, DP), since treatment initiation. The difference in survival proportions at fixed time points was estimated from Kaplan-Meier curves with corresponding 95% CIs. We further evaluated treatment effect heterogeneity through initial BI (aBI)-stratified sensitivity analyses. Statistical significance was set at *P* < 0.05.

## Results

**Fig 1:**
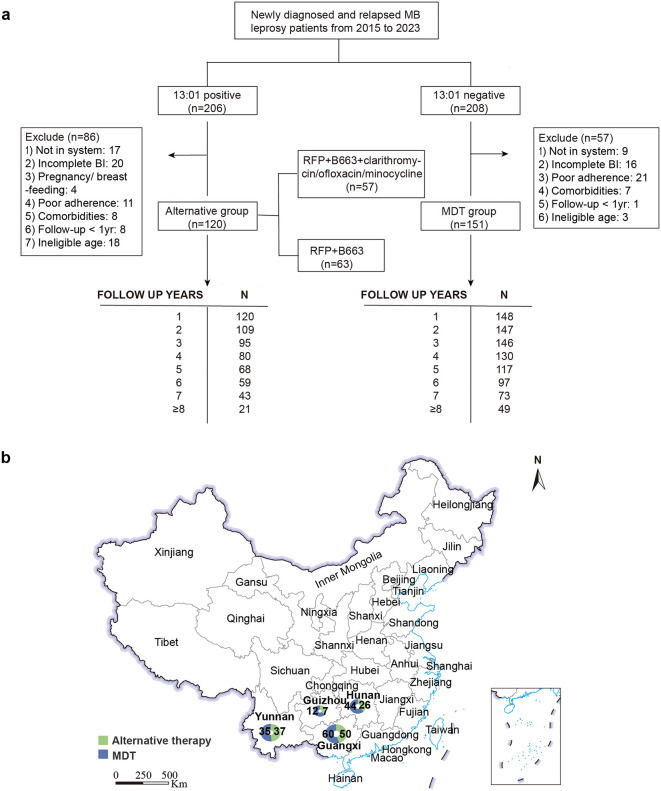
Study population screening flowchart and geographic distribution of enrolled cohorts. **(a)** Flow diagram of participant screening and enrollment (exclusion criteria detailed in Methods). **(b)** Geographic distribution of enrolled participants. The base layer is from https://www.webmap.cn/mapDataAction.do?method=forw&resType=5&storeId=2&storeName=%E5%9B%BD%E5%AE%B6%E5%9F%BA%E7%A1%80%E5%9C%B0%E7%90%86%E4%BF%A1%E6%81%AF%E4%B8%AD%E5%BF%83&fileId=BA420C422A254198BAA5ABAB9CAAFBC1 with credit to National Catalogue Service For Geographic Information.

A total of 414 participants from four Chinese provinces (Guangxi, Guizhou, Hunan, Yunnan) were enrolled through criteria-based selection, comprising an age- and sex-matched cohort with balanced HLA-B*13:01 status (206 positive vs. 208 negative). Following stringent quality control, 143 individuals were excluded, yielding a final cohort of 271 MB patients (Alternative:120; MDT:151) (Fig 1a and 1b). Participant attrition during follow-up was systematically documented in the flowchart. The cohort comprised 200 males and 71 females, with a mean age of 41.56 (±14.61) years, disease duration of 3.84 (±7.56) years, and a median follow-up period of 6.45 (4.11-8.17) years.

Baseline characteristics were comparable between the alternative therapy group and MDT group (Table 1). The distributions of key variables, including age, sex, disease duration, initial bacterial index (aBI), and clinical features (e.g., skin patches and nerve involvement), showed no significant differences. Furthermore, the median follow-up duration was 5.53 (3.21-7.80) years in the alternative group and 6.84 (5.47-8.27) years in MDT, indicating that more than half of participants in both groups were monitored for over 5.5years. Additionally, treatment completion rates reached 92.5% (111/120) in the alternative group (nine ongoing cases) versus 98.0% (148/151) in MDT, with three regimen modifications due to adverse events. The alternative protocols comprised: A. RFP + B663 (n = 57) and B. RFP + B663 + COM (n = 63).

**Table 1:** Baseline characteristics and treatment status of patients in alternative and standard multidrug therapy (MDT) groups.

Characteristic/treatment status	Total population (n = 271)	Alternative group (n = 120)	MDT group (n = 151)	*P* value
**Average age (±SD)**	41.56 (±14.61)	41.38 (±13.87)	41.71 (±15.23)	0.85
**Age groups (years)**				
0-19	22 (8.1%)	10 (8.3%)	12 (7.9%)	0.48
20-39	98 (36.2%)	42 (35.0%)	56 (37.1%)	
40-59	118 (43.5%)	57 (47.5%)	61 (40.4%)	
≥ 60	33 (12.2%)	11 (9.2%)	22 (14.6%)	
**Gender**				
Male	200 (73.8%)	85 (70.8%)	115 (76.2%)	0.32
Female	71(26.2%)	35 (29.2%)	36 (23.8%)	
**Disease duration at detection(years)**	3.84 (±7.56)	4.07 (±7.61)	3.66 (±7.53)	0.66
**BI(Mean) (±SD)**	2.96 (±1.81)	2.80 (±1.93)	3.08 (±1.71)	0.22
**BI groups**				
BI < 4	156(57.6%)	74 (61.7%)	82 (54.3%)	0.22
BI ≥ 4	115(42.4%)	46 (38.3%)	69 (45.7%)	
**Ridley Jopling Classification**				
LL	92 (33.9%)	30 (25.0%)	62 (41.1%)	0.06
BL	112 (41.3%)	54 (45.0%)	58 (38.4%)	
BB	13 (4.8%)	6 (5.0%)	7 (4.6%)	
BT	48 (17.7%)	27 (22.5%)	21 (13.9%)	
TT	6 (2.2%)	3 (2.5%)	3 (2.0%)	
**Patches**				
0-1	7 (2.6%)	4 (3.3%)	3 (2.0%)	0.78
2-5	38 (14.0%)	17 (14.2%)	21 (13.9%)	
> 5	226 (83.4%)	99 (82.5%)	127 (84.1%)	
**Nerves**				
0-1	86 (31.7%)	39 (32.5%)	47 (31.1%)	0.81
≥ 2	185 (68.3%)	81 (67.5%)	104 (68.9%)	
**Bacterial detection**				
–	36 (13.3%)	21 (17.5%)	15 (10.0%)	0.07
+	235 (86.7%)	99 (82.5%)	136 (90.0%)	
**Disability status**				
Grade 0 deformity (G0D)	195 (72.0%)	90 (75.0%)	105 (69.5%)	0.61
Grade 1 deformity (G1D)	28 (10.3%)	11 (9.2%)	17 (11.3%)	
Grade 2 deformity (G2D)	48 (17.7%)	19 (15.8%)	29 (19.2%)	
**Reaction**				
None	196 (72.3%)	89 (74.2%)	107(70.9%)	0.58
TypeⅠ	31 (11.4%)	15 (12.5%)	16 (10.6%)	
TypeⅡ	38 (14.0%)	13 (10.8%)	25 (16.6%)	
Mixed	6 (2.2%)	3 (2.5%)	3 (2.0%)	
**Treatment completion status**				
Finished	259 (95.6%)	111 (92.5%)	148 (98.0%)	
unfinished	9 (3.3%)	9 (7.5%)	0	
Treatment stopped/migrated	3 (1.1%)	0	3 (2.0%)	
**Follow-up status**				
The median duration of follow up (IQR)	6.45 (4.11-8.17)	5.53 (3.21-7.80)	6.84 (5.47-8.27)	< 0.05

### Cure and treatment duration

**Fig 2:**
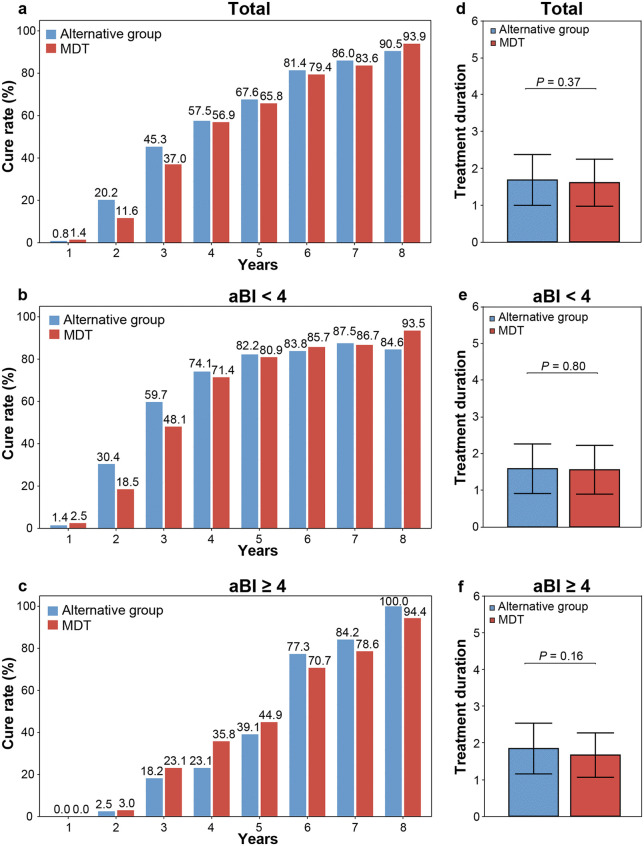
Comparison of cure rates and treatment durations between therapeutic regimens. **(a)** Annual cure rates for multibacillary (MB) leprosy patients comparing alternative therapy group with the standard MDT group. Values in data labels represent percentages (see Y-axis); the ‘%’ symbol is omitted for clarity. **(b, c)** Annual cure rates stratified by initial bacterial index (aBI; < 4 vs. ≥ 4). Cure rates were compared using Pearson *χ²* tests. **(d)** Mean treatment duration for all completers comparing alternative group with standard MDT (Overall). **(e, f)** Mean treatment duration for all completers comparing alternative group with standard MDT, stratified by aBI. Bar heights represent means, with error bars indicating standard deviations (SD). P values were calculated using two-sample t-tests.

To evaluate potential differences in clinical efficacy among treatment regimens, we initially assessed cure rates. At year 2, cure rates were 20.2% (22/109) in the alternative group versus 11.6% (17/147) in MDT; and at year 5, 67.6% (46/68) versus 65.8% (77/117). No significant between-group differences were observed at any timepoint (*P* = 0.06 and 0.80, respectively; Fig 2a and S1 Table). Additionally, we also performed Cox regression adjusting for age, sex, disease duration, aBI, and treatment regimen. Only aBI significantly influenced cure rates (HR = 0.76, *P* < 0.001) and acted as a risk factor (S2 Table). Stratified analyses by aBI (< 4 and ≥ 4; Fig 2b and 2c) and subsequent Cox regression confirmed no significant treatment effect within either strata (all *P* > 0.05; S3 Table), indicating equivalent clinical cure efficacy between alternative therapy and MDT across bacterial load levels.

We further compared treatment duration between regimens. Among completed cases, mean treatment duration was 1.68 (±0.69) years (alternative, n = 111) versus 1.60 (±0.64) years (MDT, n = 148), with no significant difference (*P* = 0.37; Fig 2d).

Stratification by aBI also revealed no intergroup disparities between groups (aBI < 4: 1.58 vs. 1.56 years, *P* = 0.80; aBI ≥ 4: 1.84 vs. 1.66 years, *P* = 0.16; Fig 2e and 2f).

### BI change

**Fig 3:**
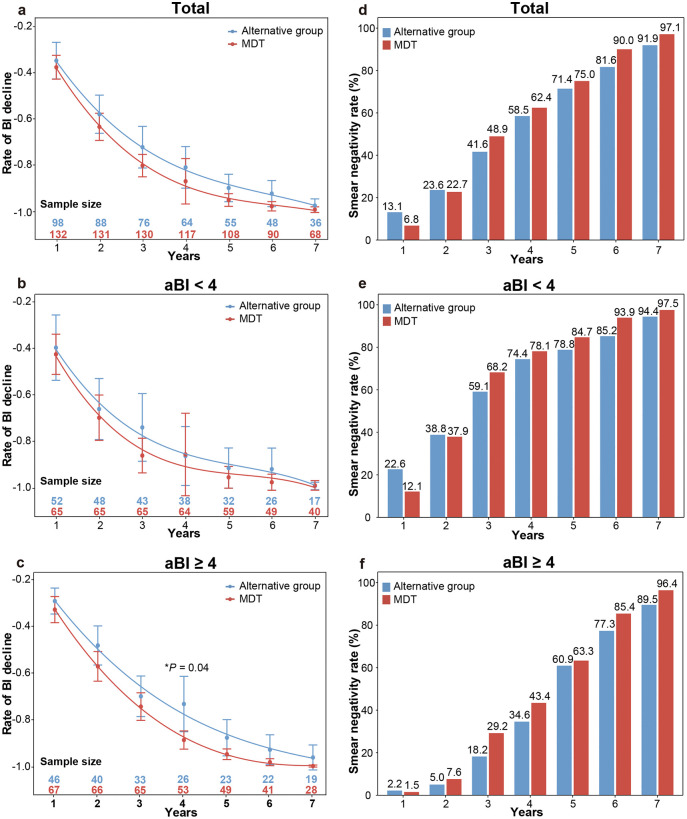
Treatment group comparisons: annual bacterial index (BI) decrease and smear negativity rates. **(a)** Mean rate of bacterial index (BI) decline from baseline to follow-up for alternative group and standard multidrug therapy (MDT). Two extreme values were excluded from analysis. Annual cohort sizes are annotated at data points. Error bars represent 95% confidence intervals. Fitting curves were generated using polynomial regression. **(b, c)** Mean rate of BI decline for alternative group and standard MDT, stratified by initial BI (aBI). **(d)** Annual smear negativity rates for multibacillary (MB) leprosy patients comparing alternative group and standard MDT (overall cohort). Values in data labels represent percentages (see Y-axis); the ‘%’ symbol is omitted for clarity. **(e, f)** Annual smear negativity rates for MB leprosy patients comparing alternative group and standard MDT, stratified by aBI.

The Bacterial Index (BI) serves as a pivotal biomarker for evaluating therapeutic efficacy in leprosy. To compare bacteriological changes between regimens, we analyzed the rate of BI decline and smear negativity rates, with annual cohort retention annotated in Fig 3. Overall, the MDT group demonstrated a more pronounced BI decline trend compared to the alternative regimen (Fig 3a), although this difference did not reach statistical significance. Specifically, no significant intergroup differences were observed at Year 2 (57.93% vs 63.39%; *P* = 0.28) or Year 5 (89.92% vs 95.11%; *P* = 0.12). Similarly, smear negativity rates did not differ significantly between groups at year 2 (23.60% vs. 22.73%, *P* = 0.88) or year 5 (71.43% vs. 75.00%, *P* = 0.62; Fig 3d).

Additionally, we further conducted sensitivity analyses stratified by aBI. Patients with aBI < 4 showed no significant differences in BI change between groups (Fig 3b and 3e). However, in the aBI ≥ 4 stratum, the MDT group generally exhibited a higher BI reduction trend than the alternative group, reaching statistical significance particularly in the 4th year, with the decrease rates of 88.64% (MDT) versus 73.22% (alternative) (*P* = 0.04), albeit marginally significant (Fig 3c). Smear negativity rates remained similar between groups in this higher-burden stratum (Fig 3f).

### Relapse condition

During follow-up, relapse rates differed numerically but not statistically between groups. The alternative group (657.53 person-years [PY]) had three relapses (year 3: one case; years 8–9: two cases), yielding a rate of 0.46/100 PY (95% CI: 0.09–1.33). The MDT group (978.68 PY) reported two relapses (both in year 3), with a rate of 0.20/100 PY (95% CI: 0.02–0.74) (S1 Table and Table 2). Poisson regression confirmed no significant difference (RR = 2.23, 95% CI: 0.37–16.95; *P* = 0.38). All five relapsed patients were male. Initial and relapse BI values are detailed in S4 Table. One MDT patient upgraded from TT to BT at relapse, while others retained initial classifications.

**Table 2:** Relapse rates during follow-up in the alternative therapy vs. the MDT group.

Treatment group	Person-years of follow-up	Relapses (n)	Relapse rate/100 person-years (95%CI)	*P* value
**Alternative therapy**	657.53	3	0.46 (0.09-1.33)	0.38
**MDT**	978.68	2	0.20 (0.02-0.74)	
**Total**	1636.21	5	0.31 (0.10-0.72)	

### Frequency of leprosy reaction and disability progression

**Fig 4:**
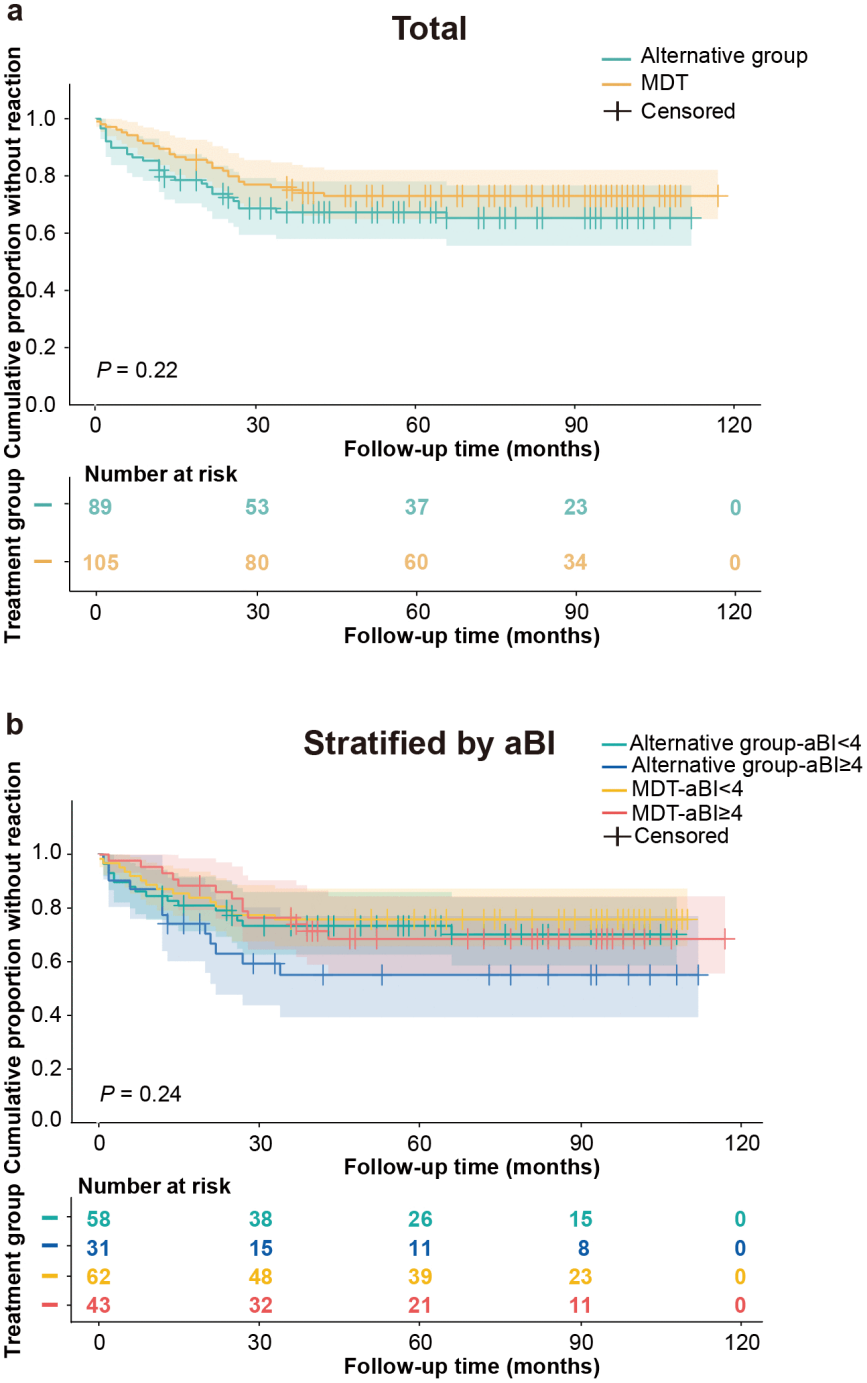
Kaplan-Meier survival curves of reaction-free multibacillary (MB) leprosy patients. **(a)** Comparison of reaction-free survival between alternative group and standard multidrug therapy (MDT). **(b)** Comparison of reaction-free survival between alternative group and standard MDT, stratified by initial bacterial index (aBI).

Survival analysis revealed no significant difference in leprosy reaction frequency between treatment regimens (log-rank *P* = 0.22). At 1 year, 89.52% of MDT-treated participants remained reaction-free, compared to 82.02% in the alternative group (risk ratio = 1.72, 95% CI [0.84–3.50]). By 3 years, reaction-free rates were 76.08% (MDT) and 67.34% (alternative), with a ratio of 1.37 (95% CI [0.86–2.16]) (Fig 4a). Stratification by aBI (< 4 or ≥ 4) also revealed no intergroup difference in reaction occurrence (*P* = 0.24) (Fig 4b).

**Fig 5:**
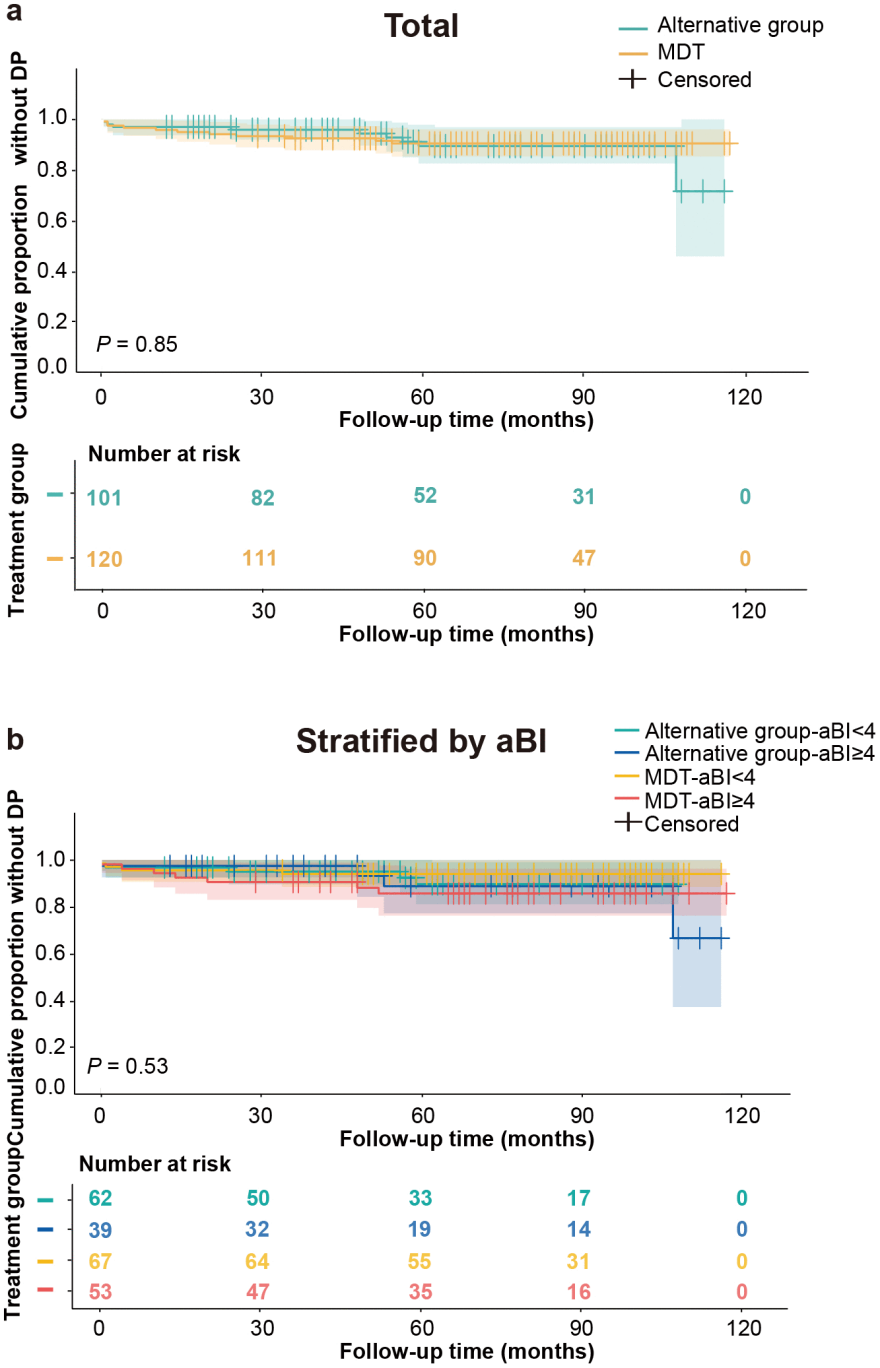
Cumulative proportion without disability progression in multibacillary (MB) leprosy patients. **(a)** Comparison between alternative group and standard multidrug therapy (MDT). **(b)** Comparison between alternative group and standard MDT, stratified by initial bacterial index (aBI).

Similarly, no significant difference was observed in overall DP between groups (*P* = 0.85, Fig 5a). By 60 months, 8.81% of alternative group patients versus 9.49% of MDT group patients had DP (risk difference [RD] = -0.68%, 95% CI [-9.04%, 7.68%]). Stratification by aBI showed no significant differences (aBI < 4: RD = 1.59%, 95% CI [-7.69%, 10.87%]; aBI ≥ 4: RD = -3.09%, 95% CI [-18.94%, 12.76%]). Only the alternative-aBI ≥ 4 group exhibited higher progression (33.4%) by 108 months, while all other subgroups remained below 15% (Fig 5b). These findings suggest comparable disability outcomes between treatment regimens.

### Adverse drug reactions

The adverse reaction rate was 1.67% (2/120) in the alternative group and 2.65% (4/151) in the MDT group, with no statistical significance (*P* = 0.70; Table 3). In the alternative group, both events were mild and did not require regimen modification, including gastrointestinal disturbances with anemia (deemed unlikely drug-related) and clofazimine-induced skin hyperpigmentation. In the MDT group, four adverse events were observed. Three were considered potentially drug-related, including transient mild pruritus resolving after treatment cessation (causative agent unspecified), elevated alanine aminotransferase (101.90 U/L; probable DDS/RFP toxicity) requiring regimen substitution with ofloxacin and clofazimine, and DDS-induced mild morbilliform rash. The remaining case (mild erythema) was judged drug-unrelated and did not necessitate treatment adjustment.

**Table 3:** Adverse drug reactions in leprosy patients receiving alternative vs. standard multidrug therapy.

No.	Time of appearance (Post-treatment initiation)	Adverse reaction phenomenon	Severity of adverse reactions	Relevance to the test drug	Suspected allergenic drugs
**1**	4 days	Gastrointestinal disturbances and anemia	Mild	Possibly irrelevant	—
**2**	3 months	Skin hyperpigmentation (reddish-brown)	Mild	Absolutely relevant	B663
**3**	——	Pruritus on the face and trunk	Mild	Possibly relevant	Uncertain
**4**	3 months	Elevated alanine aminotransferase (AAT)	Moderate	Absolutely relevant	DDS + RFP
**5**	10 months	Mild erythema	Mild	Possibly irrelevant	—
**6**	9 days	morbilliform rash	Mild	Possibly relevant	DDS

## Discussion

Dapsone potently inhibits *M. leprae* synthesis by disrupting folate metabolism [21,22]. Its inexpensive nature and role as a core component of MDT contribute to its widespread use in leprosy treatment. However, dapsone is occasionally omitted from MDT due to severe adverse effects, yet the implications for clinical efficacy and long-term relapse risks remain unclear. Therefore, we conducted a real-world exploration and found that dapsone-free alternative regimens exhibited therapeutic equivalence to standard MDT. Beyond confirming non-inferiority, our study advances the practical application of pharmacogenomics in leprosy by demonstrating that HLA-B*13:01 screening can successfully redirect carriers to effective, long-term therapy, thereby decoupling genetic risk from treatment outcome and enabling a safe care pathway for this vulnerable population. This provides substantial evidence for evaluating alternative treatment efficacy and solidifies the clinical utility of pretreatment screening.

First, we assessed the impact of standard MDT and alternative regimens on the clinical efficacy of leprosy treatment. Comparison of cure rates over an 8-year period revealed no significant differences between the groups. Corroborating our results, an Indian RCT demonstrated equivalent clinical efficacy between PB-MDT and ROM, achieving 100% cure rates by the fifth year (ROM group: 90/90, MDT group: 88/88) [12]. A recent parallel clinical trial also confirmed similar clinical and histopathological enhancements between ROM (n = 10) and MB-MDT (n = 11) [13]. Additionally, a case series of multidrug treatment with rifampicin, moxifloxacin and minocycline (RMM) in the United States reported rapid lesion clearance and definite clinical improvement in all 10 patients [23]. Meta-analyses showed no significant cure rates differences between MDT and ROM-containing regimens [24], with WHO-MDT achieving up to 99% cure rates [25]. Our longitudinal data revealed stable efficacy: at year 8, 90.5% (2 uncured) vs 93.9% (3 uncured) for alternative therapy and MDT respectively.

We also monitored the BI decline, a key indicator for prognostic assessment in leprosy [26,27]. We observed that MDT generally showed a greater BI decline trend compared to the alternative regimen, although this difference reached statistical significance only in the aBI ≥ 4 subgroup at year 4. However, the direction of this difference remained consistent in other years, suggesting it is unlikely to be a random occurrence. This may indicate that DDS within MDT confers enhanced early bactericidal activity, particularly against high bacillary loads. These findings, however, warrant further validation in larger cohorts. Importantly, both regimens achieved comparable BI reduction rates overall, reinforcing their clinical equivalence. Consistent outcomes were observed in prior smaller studies. A Nepal study of 67 dapsone-hypersensitive patients demonstrated comparable BI reduction (n = 36 smear-positive) with clofazimine-rifampicin therapy versus MDT [10]. Separately, a Guangxi 3-year study compared alternative regimens (replacing DDS with levofloxacin/clarithromycin) in HLA-B*13:01-positive MB patients with standard MDT, showing equivalent bacteriological efficacy [11]. These data indicate that both DDS-free alternative regimens and standard MDT exhibit high efficacy against *M. leprae*. Specifically, rifampicin and alternative second-line drugs (e.g., ofloxacin, clarithromycin, minocycline) demonstrate potent bactericidal activity [28–30], while clofazimine possesses dual antimycobacterial and anti-inflammatory activities, conferring sustained bactericidal effects [31]. Therefore, despite their distinct composition, DDS-free regimens still achieve effective clearance of *M. leprae* through the combined pharmacological action [32,33].

Beyond short-term efficacy, leprosy relapse remains a clinical concern. Over a maximum follow-up of 9 years, the relapse rates were comparable between alternative therapy (0.46/100 PY) and MB-MDT (0.20/100 PY). Consistent with previous research, an Indian RCT revealed non-significant relapse differences between PB-MDT (1.10/100 PY) and ROM (0.44/100 PY) over a five- to eight-year period [12]. Furthermore, no relapses were observed with either MB-MDT or ROM during ≥5-year follow-up, despite limited sample size [13]. Collectively, these findings demonstrate comparable long-term efficacy between alternative and standard regimens.

Another key concern in leprosy management is the occurrence of leprosy reactions and physical disabilities. Leprosy reactions represent acute exacerbations during the natural disease course or antimicrobial therapy, which may induce irreversible neuropathy and disability if untreated [34]. Previous studies report leprosy reaction rates of 18–65% within ≤ 3 years of multidrug therapy [14,18,35–38], while the five-year cumulative risk of disability ranges from 2.47% to 33.48% [18,39–42], with the latter variation partly attributed to differing definitions of disability outcomes across studies. In our cohort, the 3-year cumulative probabilities of reactions were 32.66% (alternative group) versus 23.92% (MDT), while 5-year disability progression occurred in 8.81% and 9.49% of patients, respectively. No significant intergroup differences emerged for either outcome, aligning with established rates—though direct comparisons of leprosy reactions and disability progression between these specific regimens remain scarce in the literature.

Finally, we also compared ADRs between two regimens and found no significant differences in safety profiles. Adverse events mainly included commonly reported reactions such as skin itching, skin pigmentation, and elevated alanine aminotransferase (ALT) levels. Noteworthy, the ADRs associated with MDT were primarily linked to DDS, consistent with previous reports. A Brazilian retrospective cohort found more ADRs in MDT than ROM (51.4% DDS-related; RFP secondary) [17], further supported by Credesh-HC-UFU data showing 37.9% MDT-associated ADRs (70.8% DDS-induced) [43]. Although no ofloxacin-associated ADRs were observed in our alternative group, a prospective study in Manaus, Brazil, reported mild ADRs in 33.3% of MB patients receiving the alternative regimen (minocycline-ofloxacin-clofazimine), with 45.9% attributed to ofloxacin (abdominal pain, nausea, headache, and insomnia) [44]. Importantly, ofloxacin increases musculoskeletal risks (e.g., tendonitis), particularly in elderly patients or those receiving corticosteroids [45].

Notably, for HLA-B*13:01-positive patients, our study demonstrates comparable efficacy between alternative regimens and conventional MDT, despite the former exhibiting slower BI decline rates, a non-significantly longer treatment duration, and higher costs of second-line alternatives (e.g., ROM regimen costs quadruple MDT expenses in the Philippines for comparable durations [13]). In contrast, for negative patients, MDT remains the preferred first-line option due to its standardized protocol, well-established efficacy, and cost-effectiveness in large-scale implementation. Accordingly, pretreatment HLA-B*13:01 screening is essential, with MDT prioritized for negative patients without allergy history.

However, this study has several limitations. First, its retrospective, non-randomized design carries potential for unmeasured confounding and selection bias. Given that pretreatment HLA-B*13:01 testing is now standard in China, this study could only compare genetically distinct groups (carriers vs. non-carriers). Although cohorts were matched for age, sex, and baseline clinical features, the HLA-B*13:01 allele may be in linkage disequilibrium with other loci that independently influence anti-*M. leprae* immune responses, disease severity, or progression, which could introduce genetic confounding. Furthermore, although standardized treatment and follow-up protocols were in place, nuanced differences in treatment adherence and inconsistencies in follow-up schedules across provinces may still exist. Second, relapse assessment was limited by insufficient follow-up and sample size, reducing statistical power. Finally, our alternative therapy was not a uniform regimen but comprised two distinct protocols. The resulting heterogeneity, along with limited subgroup sample sizes, precluded a formal inter-protocol comparison of efficacy. Consequently, prospective large-scale clinical trials and standardized treatment guidelines remain imperative.

## Conclusions

In our study, dapsone-free alternative regimens (RFP + B663 ± clarithromycin/ofloxacin/minocycline) demonstrated comparable clinical efficacy to standard MDT in MB patients and have an overall favorable safety profile, providing a viable option for HLA-B*13:01 carriers. These findings, limited by the observational design and regimen heterogeneity, warrant further investigation in prospective trials.

## Supporting information

S1 Checklist STROBE Checklist for reporting cohort studies. This checklist follows the STROBE Statement guidelines (available at https://www.strobe-statement.org/) and is used under the terms of the Creative Commons Attribution License (CC BY 4.0).
(DOCX)

S1 Text Definitions of Clinical Outcomes. Detailed criteria for cure, relapse, and disability progression adapted from WS 291–2018 with study-specific modifications.
(DOCX)

S1 Table Cure rates and relapse events in the alternative therapy vs. the MDT group during follow-up. (DOCX)

S2 Table Parametric analysis of Cox regression models for cure survival outcomes in multibacillary patients. (DOCX)

S3 Table Parametric analysis of Cox regression models for cure survival outcomes in multibacillary (MB) patients stratified by initial BI. (DOCX)

S4 Table Sociodemographic and clinical characteristics of relapsed patients during follow-up. (DOCX)

S1 Data Anonymized patient-level data and bacterial index measurements. (XLSX)
