# Supplementary material for: Outcomes of alternative therapy in HLA-B* 13:01 positive leprosy patients without dapsone versus standard MDT in negative patients: A comparative effectiveness study
Source: PLoS Negl Trop Dis. 2026 Mar 17;20(3):e0014114. doi: 10.1371/journal.pntd.0014114 (PMC13012488; doi:10.1371/journal.pntd.0014114)
Supplement: S1 Checklist — This checklist follows the STROBE Statement guidelines (available at https://www.strobe-statement.org/) and is used under the terms of the Creative Commons Attribution License (CC BY 4.0). (DOCX) [file pntd.0014114.s001.docx]

STROBE Statement—Checklist of items that should be included in reports of ***cohort studies***

|  | Item No | Recommendation | Page No | Note |
| --- | --- | --- | --- | --- |
| **Title and abstract** | 1 | (*a*) Indicate the study’s design with a commonly used term in the title or the abstract | 1 and 2 | A Comparative Effectiveness Study |
|  |  | (*b*) Provide in the abstract an informative and balanced summary of what was done and what was found | 2 | Summary in abstract |
| Introduction | | | |  |
| Background/rationale | 2 | Explain the scientific background and rationale for the investigation being reported | 4-5 | Introduction: Leprosy burden, DHS risk, *HLA-B*13:01* screening |
| Objectives | 3 | State specific objectives, including any prespecified hypotheses | 5 | Introduction: whether alternative regimens maintain equivalent efficacy |
| Methods | | | |  |
| Study design | 4 | Present key elements of study design early in the paper | 6 | The study design is presented at the beginning of the methods section. |
| Setting | 5 | Describe the setting, locations, and relevant dates, including periods of recruitment, exposure, follow-up, and data collection | 6-7 | 2015-2023, LEPMIS data, ≥1-year follow-up |
| Participants | 6 | (*a*) Give the eligibility criteria, and the sources and methods of selection of participants. Describe methods of follow-up | 6 and 8-9 | Methods: Study design; Inclusion/exclusion crieria |
|  |  | (*b*) For matched studies, give matching criteria and number of exposed and unexposed | 6 | Age- and sex-matched negative controls |
| Variables | 7 | Clearly define all outcomes, exposures, predictors, potential confounders, and effect modifiers. Give diagnostic criteria, if applicable | 7-8 | Methods: Outcome definitions and measurements |
| Data sources/ measurement | 8* | For each variable of interest, give sources of data and details of methods of assessment (measurement). Describe comparability of assessment methods if there is more than one group | 6-8 | Data sources: LEPMIS and standardized questionnaire; all outcome measurements (BI, cure/relapse, ADRs, disability) defined in "Outcome definitions and measurements" (pp. 7-8); same methods for both groups. |
| Bias | 9 | Describe any efforts to address potential sources of bias | 6 and 9-10 | Age- and sex-matched; Cox regression; sensitivity analyses |
| Study size | 10 | Explain how the study size was arrived at | 8 | Methods: Sample size |
| Quantitative variables | 11 | Explain how quantitative variables were handled in the analyses. If applicable, describe which groupings were chosen and why | 9-10 | Continuous variables: mean (±SD) or median; aBI stratified as <4 vs. ≥4 |
| Statistical methods | 12 | (*a*) Describe all statistical methods, including those used to control for confounding | 9-10 | Cox regression, Kaplan-Meier, log-rank, Poisson regression |
|  |  | (*b*) Describe any methods used to examine subgroups and interactions | 10 | aBI-stratified analyses |
|  |  | (*c*) Explain how missing data were addressed | N.A. |  |
|  |  | (*d*) If applicable, explain how loss to follow-up was addressed | N.A. |  |
|  |  | (*e*) Describe any sensitivity analyses | 10 | Initial BI-stratified sensitivity analyses |
| Results | | |  |  |
| Participants | 13* | (a) Report numbers of individuals at each stage of study—eg numbers potentially eligible, examined for eligibility, confirmed eligible, included in the study, completing follow-up, and analysed | 10 | Numbers at each stage: see flowchart in Fig 1 and text on page 10. |
|  |  | (b) Give reasons for non-participation at each stage | 10 | Reasons for exclusion (n=143) listed in the flowchart. |
|  |  | (c) Consider use of a flow diagram | 10 | Flow diagram used (Fig 1, p.10). |
| Descriptive data | 14* | (a) Give characteristics of study participants (eg demographic, clinical, social) and information on exposures and potential confounders | 10-11 | Demographic, clinical characteristics; exposures (treatment) and confounders listed in Table 1 |
|  |  | (b) Indicate number of participants with missing data for each variable of interest | N.A. |  |
|  |  | (c) Summarise follow-up time (eg, average and total amount) | 10 | Summarized in Results text (p.10) and Table 1; median (IQR) provided for each group. |
| Outcome data | 15* | Report numbers of outcome events or summary measures over time | 11-14 | Cure/relapse rates, bacterial index (BI) change, leprosy reactions, disability progression, and adverse events |
| Main results | 16 | (a) Give unadjusted estimates and, if applicable, confounder-adjusted estimates and their precision (eg, 95% confidence interval). Make clear which confounders were adjusted for and why they were included | 11-13 | Unadjusted estimates (e.g., cure rates, relapse rates, HR for BI) with 95% CIs; confounders (age, sex, disease duration, aBI) adjusted in Cox model (p.11). |
|  |  | (b) Report category boundaries when continuous variables were categorized | 11 | BI grouped as "<4 vs. ≥4" |
|  |  | (*c*) If relevant, consider translating estimates of relative risk into absolute risk for a meaningful time period | 13 | Absolute risk (risk difference) was reported for disability progression |
| Other analyses | 17 | Report other analyses done—eg analyses of subgroups and interactions, and sensitivity analyses | 11-13 | Subgroup (aBI-stratified) and sensitivity analyses reported throughout Results |
| Discussion | | | |  |
| Key results | 18 | Summarise key results with reference to study objectives | 14 | Alternative regimens exhibited therapeutic equivalence to standard MDT |
| Limitations | 19 | Discuss limitations of the study, taking into account sources of potential bias or imprecision. Discuss both direction and magnitude of any potential bias | 18-19 | Retrospective design; potential confounding (genetic, adherence); limited relapse assessment; regimen heterogeneity. |
| Interpretation | 20 | Give a cautious overall interpretation of results considering objectives, limitations, multiplicity of analyses, results from similar studies, and other relevant evidence | 15-17 | Discussion, paragraphs 2-4, pp. 15-17. |
| Generalisability | 21 | Discuss the generalisability (external validity) of the study results | 18 | Results apply to MB leprosy patients in China; MDT preferred for *HLA-B*13:01*-negative patients; alternative regimens effective for carriers. |
| Other information | | | |  |
| Funding | 22 | Give the source of funding and the role of the funders for the present study and, if applicable, for the original study on which the present article is based | 19 | Funding section |

*Give information separately for exposed and unexposed groups.

**Note:** An Explanation and Elaboration article discusses each checklist item and gives methodological background and published examples of transparent reporting. The STROBE checklist is best used in conjunction with this article (freely available on the Web sites of PLoS Medicine at http://www.plosmedicine.org/, Annals of Internal Medicine at http://www.annals.org/, and Epidemiology at http://www.epidem.com/). Information on the STROBE Initiative is available at http://www.strobe-statement.org.

This checklist follows the STROBE Statement guidelines for reporting cohort studies. The STROBE checklist is available at https://www.strobe-statement.org/ and is used under the terms of the Creative Commons Attribution License (CC BY 4.0).
