# Supplementary material for: Outcomes of alternative therapy in HLA-B* 13:01 positive leprosy patients without dapsone versus standard MDT in negative patients: A comparative effectiveness study
Source: PLoS Negl Trop Dis. 2026 Mar 17;20(3):e0014114. doi: 10.1371/journal.pntd.0014114 (PMC13012488; doi:10.1371/journal.pntd.0014114)
Supplement: S1 Text — Detailed criteria for cure, relapse, and disability progression adapted from WS 291–2018 with study-specific modifications. (DOCX) [file pntd.0014114.s002.docx]

**S1 Text. Definitions of Clinical Outcomes**

**(Adapted from WS 291-2018**^[1]^ **with study-specific modifications)**

1. **Cure**: Active symptoms (active skin lesions or peripheral nerve pain and tenderness, etc.) disappeared, no leprosy reaction or neuritis, negative bacteriological tests (three consecutive times, each interval of more than three months).
2. **Relapse:** The reappearance or exacerbation of previous skin lesions, or emergence of new clinical signs, in a patient previously treated and cured with a full course of multidrug therapy (MDT), provided that at least one of the following laboratory criteria is also fulfilled.

**Laboratory examination:**

1. Skin smear examination for acid-fast bacilli (AFB), meeting one of the following criteria:

a) Bacterial density at any skin site reached ≥2+ after prior conversion to negative;

b) Reversion to positive (with presence of solidly staining bacilli) at one or more sites following documented conversion to negative.

1. Histological examination of relapse lesions, meeting either of the following:
2. Recurrence of active lepromate-specific pathological changes without significant tissue edema;
3. Pathological findings without significant tissue edema, in conjunction with a skin smear result of ≥2+ at any site;
4. Pathological acid-fast staining was positive, with the presence of solid-staining bacilli.
5. Detection of viable bacteria by mouse foot pad inoculation.

**Clinical manifestations suggestive of relapse include:**

1. The appearance of new skin lesions (e.g., macules, papules, plaques, nodules, or diffuse infiltrations) at the sites of previously resolved lesions or on new areas of the body, typically without pronounced tenderness or edema.
2. New involvement or thickening of peripheral nerve trunks.
3. The development of new neurological complications or deformities affecting the eyes, hands, feet, or face.
4. **Disability progress (DP) :** According to WHO recommendations, deformity classification was the highest grade for any part of the hand, foot, or eyes. DP was defined as the occurrence of a new disability or the increase of WHO disability grade during treatment, which was used to compare neurological impairment between the two treatment groups.

[1] National Health and Family Planning Commission of the People's Republic of China. Diagnosis of Leprosy (WS 291-2018). Beijing: National Health and Family Planning Commission of the People's Republic of China; 2018.
