# Supplementary material for: Outcomes of alternative therapy in HLA-B* 13:01 positive leprosy patients without dapsone versus standard MDT in negative patients: A comparative effectiveness study
Source: PLoS Negl Trop Dis. 2026 Mar 17;20(3):e0014114. doi: 10.1371/journal.pntd.0014114 (PMC13012488; doi:10.1371/journal.pntd.0014114)
Supplement: S1 Table — (DOCX) [file pntd.0014114.s003.docx]

**S1 Table. Cure rates and relapse events in the alternative therapy vs. the MDT group during follow-up.**

| **Follow up (y)** | **Alternative therapy (n=120)** | | | **MDT(n=148)** | | | ***P* value** |
| --- | --- | --- | --- | --- | --- | --- | --- |
|  | **N** | **Cure number (rate)** | **relapses (n)** | **N** | **Cure number (rate)** | **relapses (n)** | **cure rate** |
|  |  |  |  |  |  |  |  |
| **1** | 120 | 1 (0.8%) | 0 | 148 | 2 (1.4%) | 0 | 1.00 |
| **2** | 109 | 22 (20.2%) | 0 | 147 | 17 (11.6%) | 0 | 0.06 |
| **3** | 95 | 43 (45.3%) | 1 | 146 | 54 (37.0%) | 2 | 0.20 |
| **4** | 80 | 46 (57.5%) | 0 | 130 | 74 (56.9%) | 0 | 0.94 |
| **5** | 68 | 46 (67.6%) | 0 | 117 | 77 (65.8%) | 0 | 0.80 |
| **6** | 59 | 48 (81.4%) | 0 | 97 | 77 (79.4%) | 0 | 0.76 |
| **7** | 43 | 37 (86.0%) | 0 | 73 | 61 (83.6%) | 0 | 0.72 |
| **≥8** | 21 | 21 (100%) | 2 | 49 | 46 (93.9%) | 0 | 0.55 |
| **Total** | — | — | 3 | — | — | 2 | — |
