# Supplementary material for: Outcomes of alternative therapy in HLA-B* 13:01 positive leprosy patients without dapsone versus standard MDT in negative patients: A comparative effectiveness study
Source: PLoS Negl Trop Dis. 2026 Mar 17;20(3):e0014114. doi: 10.1371/journal.pntd.0014114 (PMC13012488; doi:10.1371/journal.pntd.0014114)
Supplement: S2 Table — (DOCX) [file pntd.0014114.s004.docx]

**S2 Table. Parametric analysis of Cox regression models for cure survival outcomes in multibacillary patients.**

| **Variables** | **Multivariable Cox regression** | | |
| --- | --- | --- | --- |
|  | **Hazard Ratio** | **95% CI** | ***p* value** |
| Gender(female vs. male) | 0.948 | 0.689-1.302 | 0.740 |
| Age | 0.991 | 0.981-1.001 | 0.093 |
| Disease duration at detection | 0.988 | 0.967-1.009 | 0.260 |
| Initial BI | 0.758 | 0.700-0.820 | <.001 |
| Treatment grouping (Alternative group vs. MDT) | 1.147 | 0.864-1.521 | 0.343 |
