# Supplementary material for: Outcomes of alternative therapy in HLA-B* 13:01 positive leprosy patients without dapsone versus standard MDT in negative patients: A comparative effectiveness study
Source: PLoS Negl Trop Dis. 2026 Mar 17;20(3):e0014114. doi: 10.1371/journal.pntd.0014114 (PMC13012488; doi:10.1371/journal.pntd.0014114)
Supplement: S3 Table — (DOCX) [file pntd.0014114.s005.docx]

**S3 Table. Parametric analysis of Cox regression models for cure survival outcomes in multibacillary (MB) patients stratified by initial bacterial index (aBI).**

| **aBI** | **Variable** | **Multivariable Cox regression** | | |
| --- | --- | --- | --- | --- |
|  |  | **Hazard Ratio** | **95% CI** | ***p* value** |
| aBI < 4 | Gender(female vs. male) | 1.055 | 0.706-1.576 | 0.795 |
|  | Age | 0.993 | 0.981-1.006 | 0.288 |
|  | Disease duration at detection | 0.995 | 0.967-1.023 | 0.716 |
|  | Treatment regimen (Alternative group vs. MDT) | 1.197 | 0.843-1.700 | 0.314 |
| aBI ≥ 4 | Gender(female vs. male) | 1.114 | 0.677-1.834 | 0.670 |
|  | Age | 0.983 | 0.966-1.001 | 0.065 |
|  | Disease duration at detection | 1.005 | 0.974-1.036 | 0.770 |
|  | Treatment regimen (Alternative group vs. MDT) | 0.933 | 0577-1.509 | 0.778 |
