# Supplementary material for: Outcomes of alternative therapy in HLA-B* 13:01 positive leprosy patients without dapsone versus standard MDT in negative patients: A comparative effectiveness study
Source: PLoS Negl Trop Dis. 2026 Mar 17;20(3):e0014114. doi: 10.1371/journal.pntd.0014114 (PMC13012488; doi:10.1371/journal.pntd.0014114)
Supplement: S4 Table — (DOCX) [file pntd.0014114.s006.docx]

**S4 Table. Sociodemographic and clinical characteristics of relapsed patients during follow-up.**

| **Age at diagnosis (years)** | **Gender** | **Date of treatment start (month/year)** | **Ridley Jopling Classification  at diagnosis** | **Cure date (month/year)** | **Relapse Date (month/year)** | **R-J Classification at relapse** | **Initial BI** | **BI at Relapse** |
| --- | --- | --- | --- | --- | --- | --- | --- | --- |
| 41 | Male | 03/2017 | BT | 12/2018 | 10/2019 | BT | 0.1 | 0 |
| 57 | Male | 09/2015 | BT | 11/2017 | 03/2024 | BT | 0 | 0 |
| 27 | Male | 07/2015 | BL | 12/2020 | 07/2024 | BL | 5.2 | 3.3 |
| 32 | Male | 05/2015 | BT | 11/2016 | 05/2018 | BT | 0 | 0 |
| 28 | Male | 05/2015 | TT | 12/2015 | 04/2018 | BT | 0.2 | 0 |
